# The associations between sleep problems and pain outcomes in people with hand osteoarthritis – Data from the Nor-hand study

**DOI:** 10.1016/j.ocarto.2025.100579

**Published:** 2025-02-05

**Authors:** Daniel H. Bordvik, Pernille Steen Pettersen, Marthe Gløersen, Elisabeth Mulrooney, Tuhina Neogi, Ingvild Kjeken, Ida K. Haugen

**Affiliations:** a Center for Treatment of Rheumatic and Musculoskeletal Diseases (REMEDY), Diakonhjemmet Hospital, Oslo, Norway; b Oslo Metropolitan University, OsloMET, Faculty of Health Sciences, Institute of Rehabilitation Sciences and Health Technologies, Oslo, Norway; c Rehabilitation West and The Norwegian Women's Public Health Association Haugesund, Haugesund, Norway; d Section of Rheumatology, Boston University Chobanian & Avedisian School of Medicine, USA

**Keywords:** Hand osteoarthritis, Pain, Pain sensitization, Sleep problems

## Abstract

**Objective:**

To examine the relation of sleep problems to pain outcomes in people with hand osteoarthritis, and the extent to which central sensitization mediates these relationships.

**Design:**

In total 299 participants from the Nor-Hand cohort study rated their sleep problems (no, slight, moderate or severe problems), hand pain intensity on a Numeric Rating Scale (NRS, range: 0–10) and Australian/Canadian Osteoarthritis Hand Index (AUSCAN; range: 0–20), and overall bodily pain intensity (NRS). Central sensitization was evaluated by quantitative sensory testing. All pain questionnaires were repeated after 3.5 years. We explored the associations between sleep problems at baseline and pain outcomes at baseline and follow-up and fitted natural effect models to examine the extent to which measures of central sensitization mediated the effects of sleep problems on pain. All main analyses were adjusted for age, sex, education, comorbidities, and body mass index.

**Results:**

Slight, moderate, and severe sleep problems were reported by 33.8 ​%, 26.8 ​% and 14.3 ​%, respectively. In general, individuals with severe versus without sleep problems reported relatively more intense pain at baseline and follow up (i.e., a 1.68 (95 ​% confidence interval 0.89–2.46) higher NRS hand pain at baseline). Associations between sleep and central sensitization were weak, with no mediating effects found. For example, the indirect effect of remote pressure pain thresholds was 0.06 (−0.27, 0.39) NRS points for hand pain among individuals reporting severe sleep problems.

**Conclusion:**

Sleep problems are commonly reported and related to pain intensity in people with hand osteoarthritis, while the underlying mechanisms and temporal relationship remain unclear.

## 1 Introduction

Pain is a central symptom of hand osteoarthritis (OA), with an intensity ranging from mild to severe [1]. Among patients, pain is the priority symptom to improve [2,3], as chronic pain impairs quality of life and leads to seeking health care [3,4]. Pain is likely best understood in a biopsychosocial framework, in which other factors such as a person's societal characteristics, cognitions and emotions may play a role in addition to biological factors including OA pathology, genetics, central sensitization, and sleep [1,5,6]. However, the evidence exploring pain in a biopsychosocial framework in hand OA is limited.

Sleep is a basic regulated drive cardinal to human survival, development, and bodily homeostasis [7], but still there is limited research exploring the association between sleep and pain in OA [8]. Sleep problems, such as prolonged sleep onset latency, wake after sleep onset and/or premature morning awakenings, are commonly reported by people with hand OA [9,10]. In a general population cohort, the odds of frequent sleep problems were roughly 30% higher across OA patients, including persons with hand OA, compared to controls [10]. A few studies of people with knee OA report that sleep, and pain strongly associate and mutually exacerbate each other [11,12]. However, recent reviews support that sleep problems may be stronger predictors of pain than vice versa [[13], [14]]. We have less knowledge about the prevalence of sleep problems and their associations with pain outcomes in people with hand OA.

The mechanisms by which sleep problems impact pain are currently unclear. One possible mechanism may be through central sensitization [11]. Central sensitization has been defined as “*increased responsiveness of nociceptive neurons in the central nervous system to their normal or subthreshold afferent input*” by the International Association for the Study of Pain [15] and is thought to be a key mechanism leading to nociplastic pain. Sleep problems may be a risk factor for central sensitization due to the findings that sleep affects both neuronal and non-neuronal pathways thought to underpin pain perception [14,16]. No previous studies have explored the relationship between sleep problems and central sensitization in people with hand OA despite that sleep represents a possible modifiable factor in pain management, including in OA [14]. We are furthermore not aware of any OA studies that have explored whether central sensitization can mediate the relationship between sleep problems and pain intensity.

Hence, our aim was to explore the relationships of sleep problems with pain outcomes, including self-reported intensity of hand and overall bodily pain as well as indirect measures of central sensitization in a longitudinal cohort of people with hand OA. Furthermore, we aimed to examine the extent to which central sensitization mediated the relationship between sleep problems and self-reported pain intensity.

## 2 Materials and methods

### 2.1 Study population

The Nor-Hand study is a cohort of people with hand OA with data collections in 2016-17 and 2019-21 ​at Diakonhjemmet Hospital, Oslo, Norway (https://clinicaltrials.gov, Ref. no: NCT03083548). All participants were recruited from the outpatient rheumatology clinic, or a multidisciplinary course organized by the hospital. Protocols describing the two data collections and inclusion/exclusion criteria have been published [17,18]. The Norwegian Regional Committee for Medical and Health Research Ethics approved the study (Ref.no: 2014/2057 and 2019/363) and all participants provided written informed consent prior to inclusion. We involved a patient research partner in all parts of the study, including planning of design and variables, and interpretation and dissemination of results.

At baseline, we included n ​= ​300 participants aged 40–70 years, with OA in at least one finger joint or thumb base joint by ultrasound and/or clinical examination. Only participants attending both baseline and the follow-up examinations were used in longitudinal analyses. People with systemic inflammatory joint disease or psoriasis were excluded.

### 2.2 Data collection

#### 2.2.1 Sleep problems

Information on sleep status (quality and/or quantity) was obtained using the sleep component from the Norwegian 15D self-reported questionnaire [19]. Participants rated if they had *normal sleep* (no problems), *slight problems* (e.g. difficulty in falling asleep, or sometimes waking at night), *moderate problems* (e.g. disturbed sleep, or feeling I have not slept enough), *severe problems* (e.g. having to use sleeping pills often or routinely, or usually waking at night and/or too early in the morning) or *extreme sleeplessness* (e.g. sleep is almost impossible even with full use of sleeping pills, or staying awake most of the night). This tool was evaluated and used in the Oslo rheumatoid arthritis study [20] and could be used as an independent explorative screening tool [19].

Use of sleep medications was collected by asking the participants to report their regular and on-demand medications.

#### 2.2.2 Self-reported pain outcomes

Hand pain and overall bodily pain intensity during the last 24 ​h were acquired at baseline and follow-up using self-reported numeric rating scales (NRS) (range: 0–10 with 0 ​= ​no pain and 10 ​= ​worst imaginable pain). The Patient Acceptable Symptom State threshold was set to a NRS pain score ≥4 [21]. Hand pain during rest and activities throughout the last 48 ​h were obtained with the Australian/Canadian Osteoarthritis Hand Index (AUSCAN) [22], which includes 5 questions each rated on a 0–4 scale (total range: 0–20, with higher scores reflecting higher degree of pain).

#### 2.2.3 Quantitative sensory testing

QST was used as proxy measurements of central sensitization and performed by two trained medical students following the same procedures as previously published [17]. Mechanical temporal summation (TS) was measured at the left distal radioulnar joint, and pressure pain thresholds (kg/cm^2^) was measured at the midportions of the tibialis anterior muscle with a handheld digital algometer (FPIX25 Wagner, 1 ​cm^2^ flat rubber probe).

For mechanical TS, seven punctuate probes with increasing weights (8, 16, 32, 64, 128, 256 and 512 ​mN (mN)) were applied consecutively, starting with the lightest. The participant was in a sitting position with the arm rested on a table. The first probe to evoke a NRS pain ≥4 was selected for the main testing. The 512 ​mN probe was used if none of the probes provoked the necessary pain. The selected probe was tapped once per second for 10 ​s (1 ​Hz) at the left distal radioulnar joint. During testing, the participants rated their NRS pain at the first, fifth and tenth tap, and TS-delta was calculated by subtracting the rating of the first tap from the peak of the two other ratings. TS clinically translates to pain hypersensitivity or central sensitization in spinal neurons to repeated and frequency-dependent nociceptive signals, which in animals is known as the “wind-up” phenomenon [23].

PPT was assessed at one remote site relative to the hands (the midportions of the tibialis anterior muscle) to assess the presence of widespread hypersensitivity – another marker of central sensitization [24]. With the participant in a supine position, the algometer was placed perpendicular on the muscle before the pressure was increased with 0.5 ​kg/s, guided by a metronome. The participants were asked to indicate the point at which the pressure first changes to slight pain and this pressure value was noted. PPT was obtained by calculating the mean of three consecutive tests values obtained with 30-s rest periods between each measure.

Inter-examiner reliability for both PPT at the tibialis anterior muscle and TS-delta was tested on nine randomly selected participants examined by both examiners on the same afternoon at baseline. The inter-examiner reliability was moderate [25] for PPT and TS-delta with an intraclass correlation coefficient (ICC, two-way mixed-effects model with absolute agreement, individual measure) of 0.43 and 0.56, respectively.

#### 2.2.4 Clinical characteristics and confounders

Weight (in light clothing) and height (without shoes) were measured in kilograms and centimetres respectively (one decimal). Body mass index was calculated (kg/m^2^). Comorbidities were self-reported by using the comorbidity index by Sangha et al. with a sum score ranging from 0 to 45 [26]. Participants reported their highest level of education on a seven-point scale (1–7). Age and sex were obtained from medical records. The degree of anxiety/depression and pain catastrophizing was reported by the participants by completing the Hospital Anxiety and Depression Scale [27] (HADS, range: 0–42) and Pain Catastrophizing Scale [28] (PCS, range: 0–52), respectively. Self-efficacy regarding the perceived ability of an individual to influence his/her pain was measured by the Arthritis Self-efficacy score [29] (ASES) (range: 10–100). Posteroanterior radiographs of bilateral hands were obtained at both timepoints, and the distal and proximal interphalangeal, metacarpophalangeal, first carpometacarpal and scaphotrapeziotrapezoidal joints (n ​= ​32) were scored by one experienced reader (IKH) for OA severity on a 0–4 scale using a modified [30] Kellgren-Lawrence (KL) scale. We calculated a radiographic OA sum score for each participant (range 0–128). Year of symptom debut and diagnosis of hand OA were self-reported.

### 2.3 Statistical analyses

Frequency proportions, means with standard deviation (SD) or medians with interquartile range were used as appropriate to present the characteristics of the study population. We used independent t-tests, Mann-Whitney U tests or Chi-square tests to assess for differences in baseline characteristics between those with versus without follow-up data.

Due to sex differences in pain sensitivity [31] we created sex standardized PPT and TS values separately for men and women. We subtracted the sex-specific mean from each individual's observed value before dividing by the sex-specific SD. The results show a value representing the number of SDs from the average. For example, a value of 1 indicates that it is one SD away from the mean for the respective sex. All analysis were repeated by using the raw data for comparison in sensitivity models.

We assessed the cross-sectional associations between sleep problems and the pain outcomes at baseline by using separate linear regression models (Supplemental Fig. 1A and 1B). Our analyses included participants with available data on sleep at baseline and at least one baseline pain outcome, i.e. either an NRS or AUSCAN pain response or QST measure. We similarly examined whether baseline sleep problems were associated with self-reported joint pain at the follow-up examination. In these longitudinal analyses, participants with available data on baseline sleep and self-reported pain at follow-up (either NRS or AUSCAN) were included. Regression assumptions of relationships, residuals and distributions were assessed graphically by i.e., QQ-plots, boxplots and scatterplots, and multicollinearities were tested.

We explored whether baseline central sensitization mediated the relationships between sleep problems and self-reported pain intensity at baseline and follow-up by using the counterfactual framework for causal inference [32] (Supplemental Fig. 1C). In these analyses, we fitted linear natural-effects models, decomposing the effects of baseline sleep problems on pain into *indirect* (potentially mediated by central sensitization) and *direct* effects. Depending on the collected values, these estimations contrast an individual's *observed* mediator and/or outcome values with those they *potentially* would have had if counterfactually exposed (i.e., sleep problems) or not (i.e., no sleep problems). For example, subtracting the pain outcome an individual *with* sleep problems would have had if experiencing the mediator value associated with *not* having sleep problems, from that observed [32]. In all analyses, we thus assumed that sleep problems preceded central sensitization which in turn led to pain to be able to test our hypothesis [13,14]. The final estimates are presented per category of sleep problem compared to having no sleep problem with adjacent 95 ​% confidence-intervals inferred by bootstrapping (200 replicates). We also performed sensitivity analyses using the non-standardized QST values.

Due to prior assumptions of relevance to both exposures and outcomes, all analyses were adjusted for age, sex, BMI, comorbidities, and education. Due to uncertainty about casual directionality between the severity of anxiety/depression, pain catastrophizing and self-efficacy with exposures, mediators, and outcomes, we included these three variables as confounders in sensitivity analyses only. Although missing data were less than 5 ​% for each confounder, we replaced cases of missing data by simple imputations of the respective variables’ mean value (Table 1). Stata/CE v.18 basic edition, an upgrade allowing for causal mediation analyses directly from the menus, was used for all analyses.

**Table 1:** Characteristics of study participants at baseline (N ​= ​299).

Characteristic	Value
Age, mean (SD) years	60.7 (6.2)
Sex, n (%) women	265 (88.6)
Higher education and/or university, n (%)^a^	174 (58.0)
Body Mass index, mean (SD) kg/m^2^	26.5 (5.0)
Symptom duration, median (IQR) years	6 (3–13)
Years since diagnosis, median (IQR)	1 (0–6)
Hand OA by the clinical ACR criteria, n (%)	277 (92.6)
Comorbidity sum score, mean (SD) (range: 1–45)	7.7 (4.3)
KL-sum score, mean (SD) (range 0–128)	30.3 (19.2)
HADS total sum score, median (IQR) (range: 0–42)^a^	6 (3–10)
PCS total sum score, median (IQR) (range: 0–52)^a^	9.0 (5–15)
ASES, mean (SD) (range 10–100)^a^	64.1 (22.9)
Use of sleeping pills regularly or if needed, n (%)	20 (6.7)
Daily use of pain killers, No (%)	58 (19.4)
Sleep problems^b^	
• No sleep problems, n (%)	76 (25.4)
• Slight sleep problems, n (%)	101 (33.8)
• Moderate sleep problems, n (%)	79 (26.3)
• Severe sleep problems, n (%)	43 (14.3)
NRS hand pain, mean (SD) (range: 0–10)^c^	3.8 (2.3)
NRS pain in overall body, mean (SD) (range: 0–10)^c^	4.0 (2.3)
AUSCAN hand pain, mean (SD) (range: 0–20)	8.2 (4.0)
Temporal summation, median (IQR)^c^	1 (0–2)
PPT at the tibialis anterior muscle, mean kg/cm^2^ (SD)^c^	5.5 (2.6)

OA ​= ​osteoarthritis; IQR ​= ​interquartile range; NRS = Numerical Rating Scale; AUSCAN = Australian/Canadian Osteoarthritis Hand Index; ACR = American College of Rheumatology; PPT ​= ​pressure pain threshold; HADS = Hospital Anxiety and Depression Scale; PCS = Pain Catastrophizing Scale; ASES = Arthritis self-efficacy scale; PPT ​= ​pain pressure threshold.

Missing values.

Persons with missing values for outcome or exposure were not included in the analyses, while missing values for confounders were all imputed by sample means.

a *Covariates*: Education (n = 1); HADS (n = 9); PCS (n = 4); ASES (n = 3).

b *Exposure*: Sleep (n = 1).

c *Outcomes*: NRS hands (n ​= ​1); NRS overall body (n ​= ​2); temporal summation (n ​= ​2); PPT tibialis anterior (n ​= ​9).

## 3 Results

### 3.1 Patient characteristics

In total, 300 individuals were enrolled at baseline (2016–2017) in the Nor-Hand study cohort (Supplemental Fig. 2). Our cross-sectional analyses included 299 participants since one participant had not completed the question about sleep problems at baseline. Among these, 213 participants completed both visits (2019–2021) and were included in the longitudinal analyses (Supplemental Fig. 2). The mean time to follow-up was 3.5 years (range: 2.4–4.2 years). The amounts of missing data for other variables were generally low and considered to be present completely at random (Table 1).

At baseline, the mean age was 60.8 years, and the majority were women. The levels of anxiety/depression and pain catastrophizing were in the lower ranges of the scales. More than half of the sample (n ​= ​151, 50.5 ​%) reported hand pain equal to or above the Patient Acceptable Symptom State score ≥4 for NRS pain and 119 (40.2 ​%) participants had moderate to severe sleep problems. Twenty (6.7 ​%) participants reported usage of sleeping pills on either regular or occasional basis. As only one person reported extreme sleep problems (category 5) at baseline we merged this category with the category below (category 4). Except from a higher proportion reporting slight sleep problems and fulfilment of the ACR hand OA criteria among those completing both visits, demographic characteristics of participants with (n ​= ​213) versus without (n ​= ​86) follow-up data were not statistically significantly different (Supplemental Table 1).

### 3.2 Associations between sleep problems and pain at baseline

Compared to people with good sleep, people with sleep problems had more intense pain in the hands and overall body (Supplemental Table 2), with the strongest associations observed for those reporting severe sleep problems (Table 2). Sensitivity adjustments diminished the NRS pain difference between individuals with no and severe sleep problems to around 1 point, while the impact of these adjustments was stronger for AUSCAN pain (Supplemental Table 3).

**Table 2:** The associations between sleep problems at baseline and pain outcomes adjusted for age, sex, body mass index, education, and comorbidities.

	Pain outcomes at baseline Estimated differences (95 ​% CI)			Pain outcomes at follow up Estimated differences (95 ​% CI)		
Sleep problems at baseline	**NRS hands** (range: 0–10) n ​= ​298	**NRS all body** (range: 0–10) n ​= ​296	**AUSCAN** (range: 0–20) n ​= ​299	**NRS hands** (range: 0–10) n ​= ​211	**NRS all body** (range: 0–10) n ​= ​212	**AUSCAN** (range: 0–20) n ​= ​210
None	*0.00 (ref.)*	*0.00 (ref.)*	*0.00 (ref.)*	*0.00 (ref.)*	*0.00 (ref.)*	*0.00 (ref.)*
Slight	0.28 (−0.33, 0.89)	0.24 (−0.39, 0.86)	−0.08 (−1.23, 1.07)	0.13 (−0.60, 0.86)	0.30 (−0.44, 1.04)	0.15 (−0.10, 0.41)
Moderate	0.70∗ (0.02, 1.37)	0.79∗ (0.09, 1.49)	0.22 (−1.06, 1.49)	0.57 (−0.26, 1.39)	0.38 (−0.46, 1.23)	0.29 (−0.01, 0.56)
Severe	1.68∗ (0.89, 2.46)	1.59∗ (0.77, 2.39)	2.02∗ (0.54, 3.5)	1.46∗ (0.50, 2.42)	1.48∗ (0.50, 2.46)	0.61∗ (0.27, 0.95)

NRS, numeric rating scale, AUSCAN, Australian/Canadian pain subscale CI, confidence interval, Baseline 2016–17, Follow-up 2019–21. ∗ ​= ​Associations with p ​< ​0.05.

### 3.3 Associations between baseline sleep problems and pain at follow up

At follow-up, missing data on NRS hand pain, NRS pain in overall body and AUSCAN pain were present in 2, 1 and 3 participants respectively. The relationships between sleep problems at baseline and NRS pain at the follow-up examination remained comparable to the associations observed for NRS pain at baseline, while the association with AUSCAN pain at follow-up was weaker than at baseline (Table 2). Additional adjustments showed similar results (Supplemental Table 3). On a group level, pain intensity did not change much from baseline to follow-up, e.g., the mean (SD) change of NRS hand pain was −0.41 (2.20). On individual level, n ​= ​124 (59.1 ​%) of participants reported a change below the NRS minimal clinically important change value of 2 points [21]). Given these estimates, pain was deemed stable over time; therefore, analyses using change in pain as an outcome were not conducted.

### 3.4 Sleep problems and measures of central sensitization

At baseline, sleep problems were associated with lower PPTs, i.e., more sensitization. As an example, individuals with severe sleep problems had 0.45 SD lower PPT than the individuals with no sleep problems, which corresponded to 1.32 ​kg/cm^2^ (SD men ​= ​2.93) in men and 1.10 ​kg/cm^2^ in women (SD women ​= ​2.45). However, there was no clear dose-response relationship (Table 3). Additional adjustment for baseline anxiety/depression, pain catastrophizing and self-efficacy or use of non-standardized PPT-values did not change the results (Supplemental Tables 4 and 5, respectively). Less than twenty percent (N ​= ​58) reported to use any pain killers (oral or topical nonsteroidal anti-inflammatory drugs, acetaminophens or opioids/opioid-like drugs) prior to the QST (Table 1).

**Table 3:** The associations between baseline sleep problems and sex-standardized measures of central sensitization adjusted for age, sex, body mass index, education, and comorbidities.

	Measures of central sensitization at baseline	
Sleep problems at baseline	**PPT tibialis anterior muscle** Estimated difference (95 ​% CI)^a^ n ​= ​290	**TS left wrist joint** Estimated difference (95 ​% CI)^b^ n ​= ​295
None	*0.00 (ref.)*	*0.00 (ref.)*
Slight	−0.42c (−0.72, −0.11)	0.09 (−0.21, 0.38)
Moderate	−0.31 (−0.65, 0.03)	−0.01 (−0.34, 0.32)
Severe	−0.45c (−0.84, −0.05)	−0.02 (−0.40, 0.37)

PPT, pain pressure threshold; TS, temporal summation; CI, confidence interval; NRS, Numeric Rating Scale (range: 0–10).

a SD ​= ​2.45 ​kg/cm^2^ for women, and SD ​= ​2.93 ​kg/cm^2^ for men.

b SD ​= ​1.63 NRS points for women, and SD ​= ​1.15 NRS points for men. c ​= ​Associations with p ​< ​0.05.

We found no associations between sleep problems and measures of TS. Sensitivity analysis using the non-standardized values of QST assessments showed identical results (Supplemental Table 5).

### 3.5 Mediation analysis

The mediating effect of PPT at tibialis anterior was small and not statistically significant (Supplemental Table 6). As an example, the mediating effect of PPT on NRS hand pain was 0.06 (95 ​% CI -0.27, 0.39) NRS points, while the direct effect of sleep on pain was 1.58 (95 ​% CI 0.76, 2.39) for severe sleep problems. The mediating effect of TS was not explored due to lack of substantial relationships between sleep and TS.

## 4 Discussion

The Nor-Hand study is the first to explore the independent role of sleep problems on pain intensity and pain sensitization in persons with hand OA. Our findings indicated that sleep problems at baseline were associated with pain intensity in the hands and the overall body both at baseline and at the follow-up visit after 3.5 years, particularly for persons reporting severe sleep problems. The relationships between sleep problems and central sensitization were in general absent or much weaker, and no mediating effect of central sensitization was found for the relationship between sleep problems and pain.

In the current study, more than 70 ​% of the participants reported experiencing at least slight sleep problems, and 40 ​% revealed that their sleep was moderately or severely troubled. These findings are in line with previous studies reporting on the frequency of sleep problems among individuals with chronic pain conditions, including OA [33,34]. The prevalence of sleep problems in our study sample was higher than the prevalence of sleep problems registered among general populations of similar age [35] and thus aligns with a recent cross-sectional study showing that people with hand OA reported their sleep quality to be significantly lower compared to age-matched controls [9].

We found strong associations between sleep problems and pain intensity, both in the hands and the overall body. These results are comparable to previous cross-sectional studies on people with knee OA [36,37]. In addition, the relationships of sleep problems to all bodily pain in musculoskeletal conditions are well known and in line with our findings [38]. We also found that baseline sleep problems were associated with pain intensity at the follow-up examination after 3.5 years, which may be reflective of the minimal changes in pain during the follow-up period. A tendency for pain in hand OA to be stable over four years has also been shown by others [39]. In line with these observations, Lapane et al. found no significant relationships between baseline sleep and change in pain over four years in people with knee OA [36]. The observed associations between sleep problems and pain are considered clinically relevant. As an example, people with severe sleep problems reported 1.7 points higher on the 0–10 NRS for hand pain compared to people without sleep problems, which corresponds to the previously defined minimal clinically important improvement of one to two points on the NRS [21,40].

The mechanisms behind the associations between sleep problems and pain are yet to be determined [14]. Previous experimental research in *healthy* participants suggests that limiting sleep to ≤6.5 ​h per night, or fragmenting sleep (reducing its quality), could trigger low-grade inflammatory responses systemically, characterized by increased concentrations of pro-inflammatory mediators and non-neuronal cell activities within the central nervous system [11,14,16]. These responses may in turn collectively work to amplify ascending pain signals and thus contribute to elicit incident pain hypersensitivity or central sensitization [11,14,16,41]. In contrast, our results did not support that the same mechanisms are at play in people with hand OA. However, due to potential QST reliability issues, our results should be interpreted with caution. Additionally, the impact of experimental sleep deprivation on pain outcomes or pain sensitivity in healthy participants varies by exposure type (i.e., total, partial or selective sleep deprivation, or sleep fragmentation) and the subsequent modulation of either sleep quality or quantity [41], which our measure of sleep did not specifically address. On the other hand, our findings of weak associations between sleep problems and TS and remote PPT align with recent observations among people with knee OA [42,43], and the impact of experimental sleep deprivation on these outcomes in people with chronic pain remains unclear [41].

Other mechanisms linking sleep problems to pain are discussed in the literature, including for example the overlapping involvement of brain structures, opioid-activation, monoaminergic system-activities, the immune system and hypothalamus-pituitary-adrenal axis to regulate both sleep and pain, as well as mechanisms driven by circadian rhythms or psychological or cognitive stress [14,16,44]. It is thus likely that sleep and pain could affect each other bilaterally, i.e., pain may also affect sleep [12] and that pain in hand OA is multifactorial [5], with drivers beyond the joints.

In our sensitivity analyses, the associations between sleep problems and pain weakened after additional adjustment for anxiety/depression, pain catastrophizing and self-efficacy, suggesting that the associations between pain and sleep are affected by mental and cognitive factors. In line with these results, previous studies on people with knee OA have shown that low self-efficacy and higher levels of pain catastrophizing mediate the relationship between sleep problems and pain outcomes [42,45]. Finally, sleep and depression have been shown to influence pain in a complex interplay in people with knee OA [46]. Our results of a potential influence on pain from these factors may be important signals as most participants in our study reported to be in the lower scales of psychological and cognitive distress.

Clinically, both psychological and cognitive factors are considered modifiable, making them effective targets for pain prevention in OA [12,45]. Treatments for sleep, such as cognitive behavioural therapy, may also help alleviate pain in OA [47]. However, due to the unknown temporality of sleep problems and pain in our sample, we cannot suggest the potential relevance of these interventions for treating hand OA pain.

The associations of sleep problems with hand pain intensity were numerically stronger when measured by NRS than for AUSCAN, which may be explained by different focus of the two questionnaires. AUSCAN primarily assesses pain experiences during hand activities, whereas NRS encompasses the average pain over the last 24 ​h. In previous analyses of data from the Nor-Hand study, we found similar results, showing that central sensitization was more strongly associated with pain measured by NRS than for AUSCAN [48]. Activity-related pain may be affected by changes in activities of daily living to alleviate symptom burden, which may affect how the participants rate their pain and, consequently, the strengths of the associations. Furthermore, there might be different underlying pain mechanisms explaining pain during rest versus in activity.

The strengths of our study include the broad data collection including variables on central sensitization and cognitive factors in addition to sleep and pain intensity, as well as longitudinal data.

Some study limitations should be mentioned. The study design prevents us from inferring causal pathways between sleep and pain outcomes, and pain may be both a cause and consequence of sleep problems [12]. Despite potentially more severe symptoms in secondary care attendees compared to primary care, symptom severity was not an inclusion criterion in our study [17] and may enhance the generalizability of our findings beyond the current setting. Our sample was largely dominated by women, which could limit the translation of our findings to men. We further acknowledge that our question about sleep problems did not capture the multidimensional and various sleep aspects covered by more extensive tools, and the amounts of sleep problems reported in our study may thus be inaccurate. Experimental research using extended versions of the QST protocol, which includes measures of conditioned pain modulation, indicates that sleep problems may also directly impair endogenous pain inhibition capabilities in humans [14], especially in women [49]. We acknowledge that the exclusion of measures of conditioned pain modulation is a limitation of the current study. Finally, the moderate inter-examiner reliability of the QST may have introduced inconsistent measurements of central sensitization. We recognize that more extensive training among examiners prior to the study and a constraint of other examinations prior to QST could have improved our data.

In conclusion, our study demonstrates that sleep problems are common among persons with hand OA and are strongly associated with pain intensity both in the hands and the overall body, especially in people reporting severe sleep problems. The associations weakened after adjustments for emotional and cognitive factors, suggesting these factors may influence the relationship between sleep problems and pain. However, a mediating role of central sensitization was not found. Given the unknown temporalities between sleep and pain, these results should be taken with caution despite their potential clinical implications.

## Author contributions

Substantial contributions to the conception and design of the study: DHB, MG, EM, TN, IK and IKH, or acquisition/analysis of data: DHB, PSP, MG, EM, and IKH. Interpretation of data and drafting of the manuscript or revising it critically: DHB, PSP, MG, EM, IK, TN and IKH. Final approval of the publication: DHB, PSP, MG, EM, TN, IK and IKH. Agreement to be accountable for all aspects of the work in ensuring that questions related to the accuracy or integrity of any part of the work are appropriately investigated and resolved: DHB, PSP, MG, EM, TN, IK and IKH.

## Role of the funding source

The Nor-Hand study was funded by the 10.13039/501100005416Norwegian Research Council (project number: 328657), 10.13039/100027399ADVANCE grant from Pfizer/Lily, South-East Norway Regional 17 Health Authority, Pahle's foundation, Simon Fougner Hartmann's Family foundation and Trygve Gythfeldt's research foundation. TN was supported by NIH K24 AR070892, P30 AR072571 and R01 AR062506. DHB's work was completely funded by a grant from the Norwegian Women's Public Health Association's department at Haugesund Rheumatological Hospital, Haugesund, Norway. The funders were not involved in the study design, collection, analysis or interpretation of the current data.

## Conflict of interest

IKH receives consulting fees (honoraria for lectures) from 10.13039/100004336Novartis, GSK, Grünenthal and Abbvie. TN reports grants or contracts from 10.13039/100004319Pfizer/Lilly (to institution) and consulting fees from 10.13039/100004319Pfizer/Lilly, 10.13039/100009857Regeneron and 10.13039/100004336Novartis outside the current work. MG, EM, PSP and IK report nothing to disclose.
