# Supplementary material for: The associations between sleep problems and pain outcomes in people with hand osteoarthritis – Data from the Nor-hand study
Source: Osteoarthr Cartil Open. 2025 Feb 5;7(1):100579. doi: 10.1016/j.ocarto.2025.100579 (PMC11875149; doi:10.1016/j.ocarto.2025.100579)
Supplement: Multimedia component 1 [file mmc1.docx]

**Supplemental table 1:** Differences in baseline characteristics between participants attending both visits (2016/2017 and 2019-20121) compared to those participating in the baseline assessments (2016/2017) only.

| Characteristic | Values | | |
| --- | --- | --- | --- |
|  | **Baseline only**  **(n=86)** | **Both visits (n=213)** | **P-value** |
| Age, mean (SD) | 60.5 (6.6) | 60.9 (6.0) | P=0.69 |
| Sex, n (%) women | 81 (93.1) | 185 (86.9) | P=0.12 |
| Higher education and/or University, n (%) | 45 (51.7) | 129 (60.5) | P=0.16 |
| Body Mass Index, mean (SD) kg/m^2^ | 26.4 (5.4) | 26.6 (4.8) | P=0.65 |
| Symptom duration y, median (IQR) | 6 (3 – 13) | 6 (3 – 13) | P=0.79 |
| Diagnosis duration y, median (IQR) | 1 (0 – 4) | 1.5 (0 – 6) | P=0.32 |
| Fulfils ACR hand OA criteria, n (%) | 75 (87.2) | 202 (94.8) | P=0.02* |
| Comorbidity sum score, mean (SD) (range 1-45) | 8.3 (4.6) | 7.5 (4.1) | P=0.12 |
| KL sum score, mean (SD) | 27.8 (19.8) | 31.3 (18.9) | P=0.15 |
| NRS hand pain, mean (SD) (range 0–10) | 4.0 (2.4) | 3.7 (2.2) | P=0.33 |
| NRS all bodily pain, mean (SD) (range 0-10) | 4.2 (2.3) | 4.0 (2.3) | P=0.41 |
| AUSCAN pain subscale, mean (SD) | 8.7 (4.1) | 8.0 (4.0) | P=0.20 |
| No. of patients taking daily pain killers (%) | 14 (16.3) | 44 (20.6) | P=0.39 |
| Sleep problems   - No sleep problems, n (%) - Slight sleep problems, n (%) - Moderate sleep problems, n (%) - Severe sleep problems, n (%) | 22 (25.6)  22 (25.6)  27 (31.4)  15 (17.4) | 54 (25.4)  79 (37.9)  52 (24.4)  28 (13.2) | P=0.97  P=0.04*  P=021  P=0.35 |
| No. of patients taking sleeping pills (regularly or if needed) (%) | 5 (5.8) | 15 (7.0) | P=0.71 |
| Temporal summation, median (IQR- not sex std) | 1 (0 -3) | 1 (0 -2) | P=0.32 |
| PPT at the tibialis anterior muscle, mean Kg/cm^2^ (SD) | 5.5 (2.3) | 5.6 (2.7) | P=0.83 |
| HADS total sum score, median (IQR) (range 0–42) | 7 (2-12) | 6 (3-10) | P=0.32 |
| PCS total sum score, median (IQR) (range 0–52) | 9.5 (5 - 15) | 9.0 (5 -15) | P=0.93 |
| ASES, mean (SD) (range 10-100) | 63.6 (23.2) | 64.4 (22.9) | P=0.79 |

OA = osteoarthritis; IQR = interquartile range; NRS = Numerical Rating Scale; AUSCAN = Australian/Canadian Osteoarthritis Hand Index; ACR = American College of Rheumatology; PPT = pressure pain threshold; HADS = Hospital Anxiety and Depression Scale; PCS = Pain Catastrophizing Scale; ASES = arthritis self-efficacy score; PPT= pain pressure threshold. *=Differences with P<0.05.
