# Supplementary material for: The associations between sleep problems and pain outcomes in people with hand osteoarthritis – Data from the Nor-hand study
Source: Osteoarthr Cartil Open. 2025 Feb 5;7(1):100579. doi: 10.1016/j.ocarto.2025.100579 (PMC11875149; doi:10.1016/j.ocarto.2025.100579)
Supplement: Multimedia component 2 [file mmc2.docx]

**Supplemental table 2**: Characteristics of study participants with no sleep problems compared to individuals with each level of sleep problems at baseline.

| Characteristic | Sample  N=299* | No sleep problems  (ref)  n (%)  76 (25.4) | Slight sleep problems  n (%)  101 (33.8) | Moderate sleep problems  n (%)  79 (26.3) | Severe sleep problems  n (%)  43 (14.3) |
| --- | --- | --- | --- | --- | --- |
| Age, mean (SD) years | 60.7 (6.2) | 61.5 (6.0) | 61.0 (6.2) | 59.8 (6.2) | 60.4 (6.4) |
| Sex, n (%) women | 265 (88.6) | 60 (79.0) | 91 (0.90)¶ | 75 (94.9)¶ | 39 (90.7) |
| Higher education and/or university, n (%)† | 174 (58.0) | 51 (67.1) | 58 (57.4) | 45 (57.0) | 20 (46.5) |
| Body Mass Index, mean (SD) kg/m^2^ | 26.5 (5.0) | 25.5 (4.9) | 26.6 (5.2) | 27.3 (4.8) | 26.8 (4.7) |
| Comorbidity sum score, mean (SD) (range: 1-45) | 7.7 (4.3) | 5.4 (3.2) | 7.6 (4.1)¶ | 9.1 (4.2)¶ | 9.5 (4.5)¶ |
| HADS total sum score, median (IQR) (range: 0–42) † | 6 (3 – 10) | 4.7 (4.8) | 7.0 (5.7) | 9.0 (6.4)¶ | 9.5 (6.9)¶ |
| PCS total sum score, median (IQR) (range: 0–52) † | 9.0 (5 - 15) | 8.5 (7.2) | 10.4 (8.0) | 12.4 (8.1)¶ | 14.0 (8.7)¶ |
| ASES, mean (SD) (range 10-100) † | 64.1  (22.9) | 79.3  (17.2) | 66.2 (20.2)¶ | 53.3 (22.5)$¶$ | 52.3 (22.3)¶ |
| Symptom duration, median (IQR) years‡ | 6 (3 – 13) | 8.1 (8.2) | 8.5 (7.6) | 9.1 (8.0) | 10.1 (8.1) |
| Years since diagnosis, median (IQR)§ | 1 (0 – 6) | 3.5 (5.2) | 4.1 (6.4) | 3.0 (4.4) | 4.2 (4.3) |
| Hand OA by the clinical ACR criteria, n (%) | 277 (92.6) | 68 (89.5) | 94 (93.1) | 74 (93.4) | 41 (95.4) |
|  |  |  |  |  |  |
| KL-sum score, mean (SD) (range 0-128) | 30.3 (19.2) | 31.6 (18.5) | 32.5 (19.9) | 28.2 (16.5) | 26.8 (22.6) |
| Use of sleeping pills regularly or if needed, n (%) | 20 (6.7) | 1 (1.3) | 4 (4.0) | 5 (6.3) | 9 (20.9)¶ |
| Daily use of pain killers, No (%) | 58 (19.4) | 8 (10.5) | 17 (16.8) | 21 (26.6)¶ | 12 (27.9)¶ |
| NRS hand pain, mean (SD) (range: 0–10) § | 3.8 (2.3) | 2.9 (2.0) | 3.5 (2.1) | 4.1 (2.4)¶ | 5.3 (2.1)¶ |
| NRS pain in overall body, mean (SD) (range: 0-10) § | 4.0 (2.3) | 3.1 (2.0) | 3.7 (2.0) | 4.5 (2.5)¶ | 5.6 (2.1)¶ |
| AUSCAN hand pain, mean (SD) (range: 0-20) | 8.2 (4.0) | 7.3 (4.3) | 7.7 (3.8) | 8.4 (4.2) | 10.5 (3.0)¶ |
| Temporal summation, median (IQR) § | 1 (0 - 2) | 1 (0 - 2) | 1 (0 - 2) | 1 (1 - 3) | 1 (1 - 3) |
| PPT at the tibialis anterior muscle, mean Kg/cm^2^ (SD) § | 5.5 (2.6) | 6.6 (2.7) | 5.2 (2.5)¶ | 5.3 (2.3)¶ | 5.0 (2.5)¶ |

*Abbreviations*: OA = osteoarthritis; IQR = interquartile range; NRS = Numerical Rating Scale; AUSCAN = Australian/Canadian Osteoarthritis Hand Index; ACR = American College of Rheumatology; PPT = pressure pain threshold; HADS = Hospital Anxiety and Depression Scale; PCS = Pain Catastrophizing Scale; ASES = Arthritis self-efficacy scale; PPT= pain pressure threshold.

Missing values:

*= *Exposure*: Sleep (n=1)

† = *Covariates*: Education (n=1); HADS (n=9); PCS (n=4); ASES (n=3)

‡ = *Descriptive data*: Symptom duration (n=22); Years since diagnosis (n=20);

§= *Outcomes*: NRS hands (n=1); NRS overall body (n=2); temporal summation (n=2); PPT tibialis anterior (n=9)

*Note*: Persons with missing values for outcomes or exposure were not included in the analyses. Missing values for confounders were all imputed by sample means.

¶= Different from reference group (p<0.05)
