# Supplementary material for: The associations between sleep problems and pain outcomes in people with hand osteoarthritis – Data from the Nor-hand study
Source: Osteoarthr Cartil Open. 2025 Feb 5;7(1):100579. doi: 10.1016/j.ocarto.2025.100579 (PMC11875149; doi:10.1016/j.ocarto.2025.100579)
Supplement: Multimedia component 3 [file mmc3.docx]

**Supplemental table 3:** The associations between sleep problems at baseline and pain outcomes adjusted for additional confounding by anxiety/depression, pain catastrophizing and self-efficacy (comprehensive model).

|  | Pain outcomes at baseline  Estimated difference (95% CI) | | | Pain outcomes at follow up  Estimated difference (95% CI) | | |
| --- | --- | --- | --- | --- | --- | --- |
| Sleep problems at baseline | **NRS hands**  **(0-10)**  n=298 | **NRS all bodily pain**  **(0-10)**  n=296 | **AUSCAN pain**  **(0-20)**  n=299 | **NRS hands**  **(0-10)**  n=211 | **NRS all bodily pain**  **(0-10)**  n=212 | **AUSCAN pain**  **(0-20)**  n= 210 |
| None | *0.00 (ref.)* | *0.00 (ref.)* | *0.00 (ref.)* | *0.00 (ref.)* | *0.00 (ref.)* | *0.00 (ref.)* |
| Slight | -0.08  (-0.66, 0.51) | 0.01  (-0.61, 0.63) | -0.63  (-1.75, 0.49) | -0.01  (-0.73, 0.72) | 0.17  (-0.55, 0.90) | 0.10  (-0.15, 0.36) |
| Moderate | -0.01  (-0.69, 0.51) | 0.27  (-0.46, 1.00) | -0.90  (-2.20, 0.41) | 0.28  (-0.56, 1.18) | 0.07  (-0.78, 0.91) | 0.18  (-0.12, 0.47) |
| Severe | 0.91*  (0.12, 1.69) | 1.19*  (0.36, 2.03) | 0.72  (-0.7, 2.22) | 1.10*  (0.12, 2.08) | 1.11*  (0.12, 2.09) | 0.47* (0.13, 0.82) |

NRS; numeric rating scale, AUSCAN; Australian/Canadian pain subscale CI; confidence interval, Baseline 2016-17, Follow-up 2019-21. *=Associations with p < 0.05.
