# Supplementary material for: The associations between sleep problems and pain outcomes in people with hand osteoarthritis – Data from the Nor-hand study
Source: Osteoarthr Cartil Open. 2025 Feb 5;7(1):100579. doi: 10.1016/j.ocarto.2025.100579 (PMC11875149; doi:10.1016/j.ocarto.2025.100579)
Supplement: Multimedia component 4 [file mmc4.docx]

**Supplemental table 4**: The associations between baseline sleep problems and sex-standardized proxy measures of central sensitization additionally adjusted for anxiety/depression, pain catastrophizing and self-efficacy at baseline (comprehensive model).

| Sleep problems  at baseline | PPT Tibialis anterior muscle*  Estimated difference (95% CI) n=290 |
| --- | --- |
| None | *0.00 (ref.)* |
| Slight | -0.36† (-0.68, -0.05) |
| Moderate | -0.20 (-0.57, 0.17) |
| Severe | -0.33† (-0.75, -0.09) |

PPT= pressure pain threshold. *= SD=2.45 kg/m^2^ for women, and SD=2.93 kg/m^2^ for men. †= Associations with p < 0.05.
