# Supplementary material for: The associations between sleep problems and pain outcomes in people with hand osteoarthritis – Data from the Nor-hand study
Source: Osteoarthr Cartil Open. 2025 Feb 5;7(1):100579. doi: 10.1016/j.ocarto.2025.100579 (PMC11875149; doi:10.1016/j.ocarto.2025.100579)
Supplement: Multimedia component 5 [file mmc5.docx]

**Supplemental Table 5**: The associations between baseline sleep problems and non-standardized measures of central sensitization, adjusted for age, sex, body mass index, education, and comorbidities*.*

|  | Measures of central sensitization at baseline | |
| --- | --- | --- |
| Sleep problems  at baseline | **PPT tibialis anterior muscle***  Estimated difference (95% CI)  n=290 | **TS left wrist joint†**  Estimated difference (95% CI)  n=295 |
| None | *0.00 (ref.)* | *0.00 (ref.)* |
| Slight | -1.1 (-1.8, -0.3)^‡^ | 0.2 (-0.3, 0.7) |
| Moderate | -0.8 (-1.7, 0.1) | 0.0 (-0.5, 0.6) |
| Severe | -1.1 (-2.1, -0.1)^‡^ | 0.1 (-0.6, 0.7) |

PPT, pressure pain threshold; TS, temporal summation; CI, confidence interval. *=kg/m^2^; †= Numeric rating scale (range 0-10); ‡ = Associations with p < 0.05.
