# Supplementary material for: The associations between sleep problems and pain outcomes in people with hand osteoarthritis – Data from the Nor-hand study
Source: Osteoarthr Cartil Open. 2025 Feb 5;7(1):100579. doi: 10.1016/j.ocarto.2025.100579 (PMC11875149; doi:10.1016/j.ocarto.2025.100579)
Supplement: Multimedia component 6 [file mmc6.docx]

**Supplemental table 6:** Natural indirect and natural direct effects of sleep problems on pain outcomes at baseline mediated by measures of pain pressure thresholds* at the tibialis anterior muscle.

|  | NRS hand pain as outcome | | NRS all bodily pain as outcome | | AUSCAN pain as outcome | |
| --- | --- | --- | --- | --- | --- | --- |
| Sleep problems | **Indirect effect**  Beta  (95% CI) | **Direct effect**  Beta  (95% CI) | **Indirect effect**  Beta  (95% CI) | **Direct effect**  Beta  (95% CI) | **Indirect effect**  Beta  (95% CI) | **Direct**  **effect**  Beta  (95% CI) |
| None | 0.00 (ref.) | 0.00 (ref.) | 0.00 (ref.) | 0.00 (ref.) | 0.00 (ref.) | 0.00 (ref.) |
| Slight | 0.15  (-0.03, 0.33) | 0.08 (-0.46, 0.62) | 0.06 (-0.14, 0.26) | 0.17 (-0.44, 0.76) | -0.32  (-0.75, 0.11) | 0.05  (-1.28, 1.38) |
| Moderate | 0.03  (-0.15, 0.20) | 0.60 (-0.11, 1.31) | 0.06  (-0.16, 0.29) | 0.81†  (0.03, 1.59) | 0.12  (-0.24, 0.48) | -0.13  (-1.55, 1.29) |
| Severe | 0.06  (-0.27, 0.39) | 1.58 †  (0.76, 2.39) | 0.22  (-0.16, 0.60) | 1.54†  (0.80, 2.29) | -0.06  (-0.44, 0.33) | 1.86†  (0.42, 3.30) |

NRS; numeric rating scale, AUSCAN; Australian/Canadian pain subscale CI; confidence interval. Adjusted model (sex, age, comorbidities, education, and body mass index). * = mediation per sex-standardized standard deviation of pain pressure threshold values, SD =2.45 kg/m^2^ for women, and SD=2.93 kg/m^2^ for men. †=Associations with p<0.05
