# Supplementary figures and images for: The associations between sleep problems and pain outcomes in people with hand osteoarthritis – Data from the Nor-hand study
Source: Osteoarthr Cartil Open. 2025 Feb 5;7(1):100579. doi: 10.1016/j.ocarto.2025.100579 (PMC11875149; doi:10.1016/j.ocarto.2025.100579)

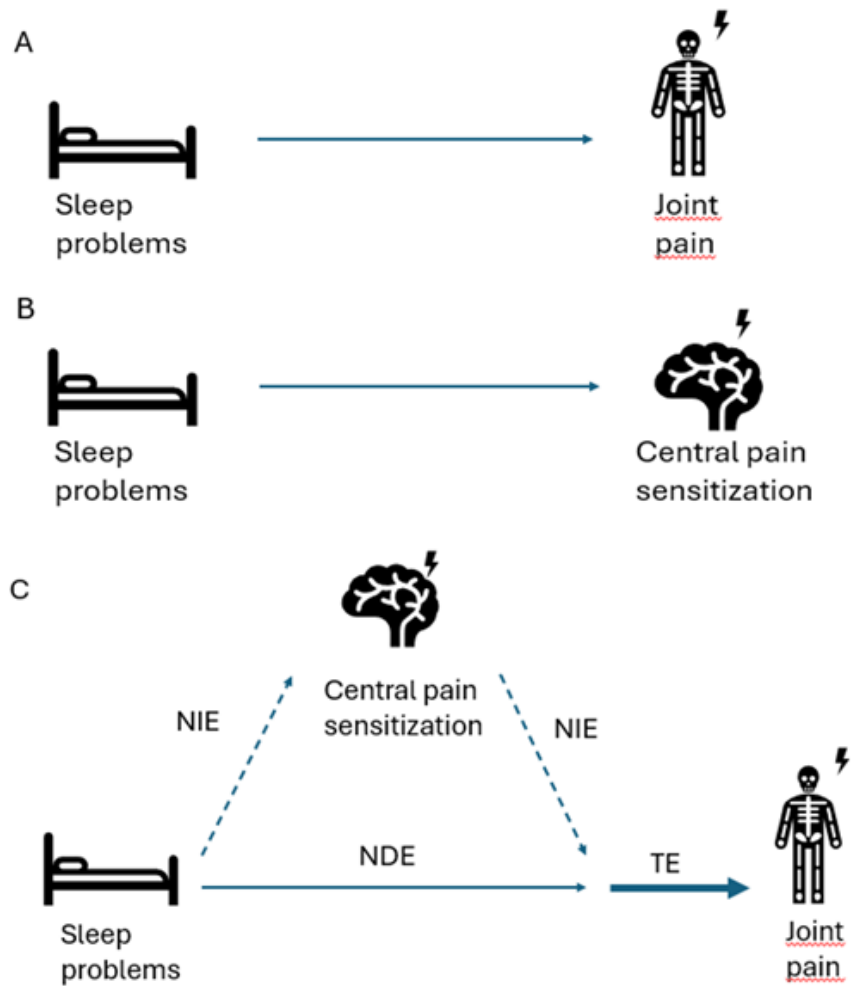

Supplement: Multimedia component 7 [file mmc7.pdf]

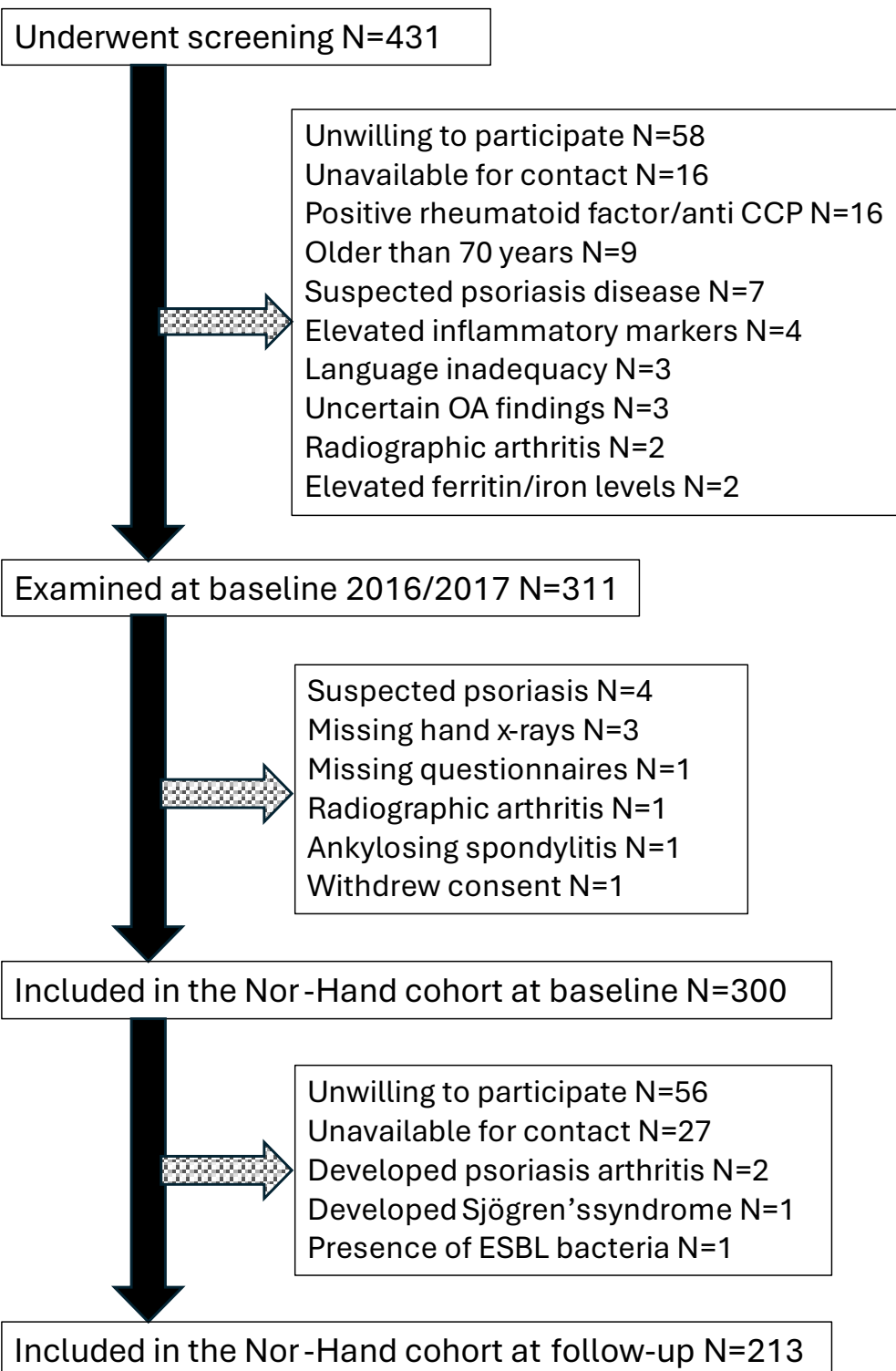

Supplement: Multimedia component 8 [file mmc8.pdf]
