# Supplementary material for: The associations between sleep problems and pain outcomes in people with hand osteoarthritis – Data from the Nor-hand study
Source: Osteoarthr Cartil Open. 2025 Feb 5;7(1):100579. doi: 10.1016/j.ocarto.2025.100579 (PMC11875149; doi:10.1016/j.ocarto.2025.100579)
Supplement: Multimedia component 9 [file mmc9.docx]

**Supplemental figure 1:** Associations between sleep problems at baseline and joint pain (**A**); Associations between baseline sleep problems and outcomes of central pain sensitization (**B**); Natural indirect effects (NIE), natural direct effects (NDE) and total effects (TE) of sleep problems on outcomes of joint pain at baseline (**C**).
