# Supplementary material for: The associations between sleep problems and pain outcomes in people with hand osteoarthritis – Data from the Nor-hand study
Source: Osteoarthr Cartil Open. 2025 Feb 5;7(1):100579. doi: 10.1016/j.ocarto.2025.100579 (PMC11875149; doi:10.1016/j.ocarto.2025.100579)
Supplement: Multimedia component 10 [file mmc10.docx]

**Supplemental figure 2**: Flowchart showing the inclusion of participants in the Nor-Hand study (*Abbreviations*: CCP, cyclic citrullinated protein; ESBL, extended spectrum beta-lactamase).
